# Economic Valuation of Ecosystem Services and Its Association with Socioeconomic Factors in the Wof-Washa Natural State Forest, North Shewa Zone, Ethiopia

**DOI:** 10.1155/2024/6607551

**Published:** 2024-07-16

**Authors:** Abere Wondimu Kassie, Admasu Moges, Tilahun Alelign, Gojam Bayeh

**Affiliations:** ^1^ Department of Statistics College of Natural and Computational Sciences Debre Berhan University, P. O. Box 445, Debre Birhan, Ethiopia; ^2^ Department of Biology College of Natural and Computational Sciences Debre Berhan University, P. O. Box 445, Debre Birhan, Ethiopia

## Abstract

The Wof-Washa Natural State Forest (WWNSF) in Ethiopia harbors remarkable biodiversity but faces threats from local communities, climate change, and a lack of awareness regarding its preservation. Although numerous studies exist, the economic value of the forest's ecosystem services (FESs) has been largely overlooked. Thus, the purposes of this study were to identify FESs, estimate their total economic value, and assess challenges with possible strategies for sustainable forest management. The research employed Google Earth to estimate forest area and socioeconomic surveys with field visits to gather data from 368 participants for analyzing the FESs, monetary values, and challenges with possible strategies. Analyses included descriptive statistics, total economic valuation (TEV), and an ordinary least squares regression model. The study revealed that the WWNSF provides crucial provisioning services (timber, honey, water, and firewood), regulating services (soil erosion control and climate change mitigation), and supporting services (soil formation and nutrient cycling). The forest's annual contribution to sampled households averaged $1152.30, with regulating and cultural services valued at $14,112 and $622.00, respectively. The study also revealed that male-headed households, larger families, and those having limited farmland and off-farm income depended more heavily on the forest. Therefore, farmland scarcity, settlements, and tree harvesting for firewood, timber, and agricultural tools, as well as the lack of employment opportunities, were the main challenges encountered in the study area. This suggests that despite degradation and reduction in size due to human pressures, the WWNSF remains a significant source of socioeconomic and ecological benefits, supporting local livelihoods and biodiversity. To ensure the forest's restoration, prioritizing community participation, promoting family planning, providing alternative income opportunities especially for youth, and balancing resource use with conservation efforts are possible strategies that should be taken into account.

## 1. Introduction

Forests are a cornerstone of our planet's health, offering immense economic, ecological, and cultural value. Recognizing this importance, global efforts began in the early 20th century to protect and manage forests effectively. These efforts aim to preserve valuable forest products (like timber), sustain ecological functions (nutrient and water cycle), and safeguard unique cultural and aesthetic landscapes [1]. Ethiopia, a biodiversity hotspot, exemplifies this need for forest conservation; its diverse landscapes boast an abundance of woody flora and contribute significantly to the Horn of Africa's natural resources [2]. These forests are vital habitats, safeguarding agricultural diversity and preserving Ethiopia's biodiversity [3]. They also provide a range of products beyond timber, including bamboo, honey, medical plants, and incense [4]. Notably, forests have the potential to contribute significantly to Ethiopia's climate change mitigation goals [3]. The benefits that humans derive from ecosystems are termed ecosystem services (ES). When provided specifically by forests, these are referred to as forest ecosystem services (FES) [5]. Forest ecosystems (FESs) are multiuse natural resources known for their potentiality in providing various ESs [6]. The ESs were introduced to raise awareness of the importance and conservation of biodiversity [7]. FESs can be categorized as provisioning, cultural, regulating, and supporting services based on their benefits to human well-being and ecology. The service areas provided by the ecosystem are also referred to as “natural capital” due to their contribution to biodiversity that benefits humans [7]. Forests are therefore the main providers of ESs, crucial for life support systems [6, 8]. The FESs offer direct (material and nonmaterial) and indirect benefits to human well-being, including raw materials, aesthetics, and cultural and ecosystem regulations [4, 9, 10].

However, the tropical forest and other vegetation cover including inland waters have declined due to agricultural expansion, population growth [6], erosion and sedimentation, traditional shifting cultivation, intensive agriculture, and urbanization [11–13]. Particularly, forests in Africa, including in Ethiopia, have faced serious deforestation, which occurs when forestland is converted to nonforested land for agriculture, grazing, or urban development [14]. According to FAO [15], deforestation in tropical forests from 1990 to 2016 was severe and extensive [15], and Ethiopia's landscape was severely degraded [6, 16]. Biru [17] reported that deforestation has resulted in reducing 40% of the country's land mass covered with forest in the beginning of the 20th century to 2.3% in 2000. This in turn has led to loss of biodiversity, degradation of soils, climate change, hydrological modification, and desertification. Despite Ethiopia's oldest proclaimed state forest which is rich in biodiversity, WWNSF is the most threatened ecosystem [18]. According to Haike et al. [19], the forest cover of WWNSF had decreased by 4.58% since 2000–2022.

Many studies have been conducted in WWNSF with respect to floristic composition [20], plant diversity and structural analysis [21], vegetation ecology and carbon stock [22], tourism potentials [23], impact of altitude and anthropogenic disturbance on plant species' diversity and structure [24], and effects of forest cover changes on ESs [6]. However, the FESs and their economic values generated by the WWNSF have not yet been studied, in particular, and even at the national level, in general. The socioeconomic factors influencing forest dependence were also not addressed. Moreover, the stakeholders responsible for the protection and use of WWNSF were unknown. Even the local communities lack understanding of the FESs, such as carbon sequestration, soil erosion control, water purification, and oxygen regulation. Rather, they perceive the forest as a wasteland, resulting in the destruction of forests with a rapid decline in their area cover over time. Therefore, it is recommended to incorporate FESs and stakeholders' evaluation into decision-making processes to make informed decisions about the benefits and costs of different policy options involving natural resources [25, 26]. Hence, the contribution of this study is to estimate the total economic value of its various services, highlighting its crucial role in supporting local livelihoods. Thereby, this study could not only demonstrate the economic value of forests' benefits but also empower us to develop strategies alongside stakeholders. This collaborative approach would allow us to restore forest ecosystems, mitigate global warming, and emphasize the critical link between a healthy forest and the well-being of our entire planet.

It is therefore timely and urgent to conduct a research for assessing the FESs and estimating their monetary values in terms of money to show the contribution of the forest to local communities in supporting their subsistence livelihoods and maintaining biodiversity, as well as for identifying the challenges and the stakeholders to design the possible strategies used to minimize the local people's pressures impacting the forest. Therefore, the objectives of this study were to: (1) identify the main FESs provided by WWNSF; (2) estimate the cost of each FES based on the current local, national, and/or global market values; (3) analyze the association between household's socioeconomic characteristics and FESs' economic values; and (4) assess stakeholders, the main challenges, and their possible strategies for sustainable forest management in the study area.

## 2. Methodology

### 2.1. Study Area

**Figure 1:**
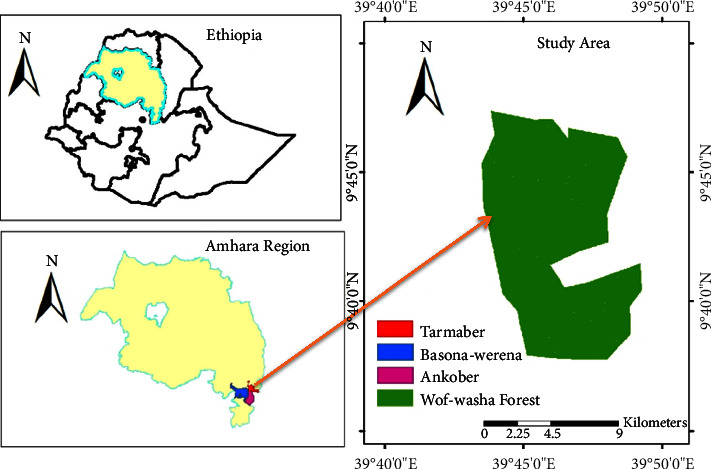
Location of the study area [6].

This research was carried out in WWNSF of the North Shewa Zone, the Amhara Regional State of central Ethiopia. It has geographic coordinates of 9°42′-9°47′ Northing and 39°43′-39°49′ Easting, located at an altitude range of 1,650–3,600 meters above sea level (masl) (Figure 1). It is bordered by the Tarmaber District in the north and northeast, the Ankober District in the south, and the Basona-Worena District in the west. The study area is characterized by tropical Afromontane cool humid, cold and humid, and cold and moist climates [6]. The forest is likely well-drained, nutrient-rich volcanic soils typical of the Ethiopian highlands. Its vegetation is likely a complex mosaic of Afroalpine and Afromontane plant communities, with potential dominance by conifers, broadleaf evergreen, and bamboo [27]. Sparse human settlements likely border the forest, with agricultural activities, potentially impacting the fringes. The mean annual temperature of the area is 13.13°C, with the mean annual minimum and maximum temperature being 6.3°C and 2°C, respectively. The rainfall in the region follows a bimodal distribution, with a mean annual rainfall of 1,840 mm.

As also reported by different documents of the agricultural offices of the Ankober and Tarmabert districts, WWNASF websites, and other scholars, there are approximately 193 species of plants in the forest, besides wild cats, monkeys, baboons, and more than half of Ethiopia's highland bird species.

### 2.2. Reconnaissance Survey and Study Design

In early November 2021, researchers conducted a preliminary visit to WWNSF to become familiar with the area and gather information from stakeholders. This visit involved estimating the total area of the WWNSF at 8,031.7 hectares, primarily located in the Tamaber (5,435 ha) and Ankober (2,596.7 ha) districts, using Google Earth survey. To collect data, a cross-sectional study design was chosen due to the one-time data collection point. Finally, researchers employed analytical and descriptive approaches to analyze the gathered data.

### 2.3. Subjects of the Respondents

The study targeted local communities, kebele (the smallest governmental administrative unit), district administrative experts, and agricultural offices of Tarmaber and Ankober districts in the North Shewa Zone. Due to budget and time constraints, and inaccessibility with insignificant influence of people of few kebeles from Basona-Worena District, the researchers were unable to survey all households of Kebeles that border the WWNSF. Instead, we used a sample and sample size determination formula to select the households for the study, which is explained in the next section. The actual data were however collected from January to May 2022.

### 2.4. Sample Size, Sampling Techniques, and Data Collection Tools

The study evaluated the economic values of FES and analyzed WWNSF stakeholders, primarily located in the Ankober and Tamaber districts since the forest area in Basona Worena is smaller and difficult to access due to its hilly terrain. Therefore, the researchers included respondents from the neighboring kebeles of the Basona-Worena District. To select respondents from four kebeles, a stratified sampling technique was used. Kebeles were first divided (stratified) based on their location relative to WWNSF (accessibility). Hence, the total of target households was 1,612. Specifically, *N*1 = 547 from the Wof-Washa kebele largely bordering the forest on the side of the Tarmber District, *N*2 = 435 from Mehal-Wonz, *N*3 = 286 from Laygorbela, and *N*4 = 344 from the Zego kebeles of the Ankober District were selected because a large size of the WWNSF is located in the Ankober District followed by Tarmaber (Table 1). Assuming that 35% of the households in each kebele had a direct impact on the forest, a 95% confidence level (*Z* = 1.96) and 6% precision, the sample size was estimated.

(1) $$\begin{array}{c} n=\frac{Z^{2}\text{pq}}{d^{2}}=\frac{{\left(1.96\right)}^{2}*0.35*0.65}{0.06^{2}}=242.77\approx 243. \end{array}$$

In addition, to account for nonparticipation in the survey, a 10% estimated sample of households was included, resulting in a total of 268 sample households. Subsequently, the sample households were selected using the simple random sampling (SRS) technique. Here, SRS is more suitable to select appropriate households which represent entire households in the community from each kebele as follows:

(2) $$\begin{array}{c} n_{1}=\frac{N_{1*n}}{N}=\frac{268*547}{1,612}=90.94\approx 91,\\ n_{2}=\frac{N_{2*n}}{N}=\frac{268*435}{1,612}=72.32\approx 72,\\ n_{3}=\frac{N_{3*n}}{N}=\frac{268*286}{1,612}=47.55\approx 48,\\ n_{4}=\frac{N_{4*n}}{N}=\frac{268*344}{1,612}=57.19\approx 57. \end{array}$$

Accordingly, as shown in Table 1, Wof-Washa Kebele of the Tarmaber District, and Laygorebela, Mehal-wonz, and Zego Kebeles of the Ankober District were the sampling sites selected for collecting the questionnaire-based data pertinent to the FES and WWNSF stakeholders. The aforementioned three kebeles of Ankober district bordered WWNSF.

In addition, 60 household leaders near WWNSF were selected for key informant interviews (KIIs). Various stakeholders including the Forest Development and Rehabilitation Program Zone Officer, District and Kebele Officers, REDD + project staff, and community members were also interviewed. We also conducted four focus group discussions (FGDs) (one per Kebele) in the two districts to better understand the community's perception of the mission. Focus group discussion (FGD) could help reveal true feelings and understanding, besides increasing our chances of understanding. Group size was ten participants. Finally, the total participants for this study were 368.

### 2.5. Methods of Data Analysis

#### 2.5.1. Descriptive Statistics and Total Economic Valuation

Different evaluation models/methods were used to assess the FESs and stakeholders in the study area, including total economic valuation (TEV) (Table 2). In addition, percentage, frequency, and content (narration) analyses were applied. Hence, for displaying the results, tables and figures were used. Therefore, the TEV was mainly use to estimate the total value of all types of FES by adding market values. The TEV is typically calculated as the sum of direct and indirect benefits provided [25, 28].

#### 2.5.2. Valuing Provisioning FESs

Structured and semi-structured questionnaires were administered to household representatives (mainly heads) to gather data on provisioning services. Local measurements such as bundle (*Esir* local name) for quantifying firewood and fodder; Jar (Jerikan) for water provision for household consumption, cattle watering, and home garden cultivation; and counting individual byproducts (like timber and farming tools) were used to quantify these services. The value of ecosystem services (ESs) was then derived by combining several factors. First, consensus information was used to determine the average amount of ESs required per household, such as the average number of livestock per household. Then, these community-specific averages were combined with average market prices for each relevant commodity. To determine market prices where no established market existed, participants were asked to reach a consensus on a theoretical selling price for a specific good.

A limitation of the data acquisition technique arose from the community's concern that the data might be used for law enforcement purposes related to forest use. For instance, although they had used timber logging for their own house construction, forest encroachment for getting new farmland and honey harvesting from the forest, they attempted to hide the products they obtained during the survey. However, to address these biases, we actively communicated and made them aware about the true objectives of the study.

#### 2.5.3. Monetary Estimates of WWNSF ES Values

The monetary values of the FES including regulating, supporting, cultural aspects, and provisioning were estimated using the standard cost estimation method ($/ha) for tropical forest areas [9, 29].

#### 2.5.4. Linear Regression Analysis

An Ordinary Least Squares (OLS) regression model was used to examine the relationship between the total annual value of provisioning FES per household (dependent variable) and various independent variables. Independent variables included characteristics of the household heads (sex, age, education, family size, land size, and off-farm activities). The standard OLS regression equation was provided as follows, explaining the role of coefficients, intercept, and residuals.

(3) $$\begin{array}{c} Y_{i}=\beta_{0}+\beta_{1}X_{1}+\beta_{2}X_{2}+\ldots +\beta_{p}X_{p}+\epsilon_{i}, \end{array}$$

*Y*_*i*_ is the dependent variable, while *X*_*i*_′*s* are independent variables. *β*_*i*_′*s* are regression coefficients, and *β*_0_ is the regression intercept; it predicts the expected value for Y if all independent variables are zero. The residual errors, *ϵ*_*i*_, in the regression equation account for the unexplained values not accounted for by the model. Multicollinearity, a potential issue where independent variables may highly correlate, was assessed using tolerance and Variance Inflation Factor (VIF) values. High VIF values indicate redundancy. In the OLS regression analysis, variables with a *p* value less than 0.05 were considered statistically significant in explaining the dependent variable (total FES value).

#### 2.5.5. Data Processing and Tools

Collected data were entered into SPSS version 25 for editing and cleaning. Cleaned data were then exported to STATA 14.2 software for statistical analysis.

## 3. Results and Discussion

### 3.1. Respondent Characterization

A total of 268 households participated in the study (summarized in Table 3). Of the total, 246 (91.8%) were men and 22 (8.2%) were women respondents. The largest age group among the respondents was 46–60 years old (42.2%), while only 7.8% were over 60 years old. Most of the respondents were literate (80.1%), with literacy levels ranging from basic reading and writing skills to completing grade 12. This implies that the majority of the respondents were literate and active participants in generating and boosting the economy of the study area. Regarding family size, 47% had six or more family members, and most households had ≤1 ha of land per family/household (85.8%). Other authors also reported similar land size per household [30–32] from Ethiopia. This indicates that land is scarce and households need to resort to additional activities or tree harvesting to provide food security for their families. Yet, only a small portion (15.3%) participated in off-farm activities such as tree seed collection, guard duties, poultry rearing, and apiculture (Table 3). Similar study also suggested those additional subsequent mitigation measures such as providing energy sources, market plantations, and conserving remnant natural forests [17].

### 3.2. Major FESs Derived from WWNSF

The FESs of WWNSF were grouped into four categories in the current study, as also reported by some other authors [30–32] and by Shiferaw et al. [6] and Muche et al. [4] for aquatic and forest ecosystems, respectively. The forest provided timber, firewood, farming tools, and water for humans and animals. The forest also offered cultural services such as recreation and traditional ceremonies, as well as regulating and supporting services such as climate regulation, soil erosion control, water and sediment retention, soil formation, nutrient cycling, and waste treatment. It also provided soil formation and habitat services for flora and fauna. Besides conducting the survey for collecting and identifying the plant specimens from the forest, other scholars [20, 22] reported various plant species found in WWNSF (Supplementary file (available here)). Moreover, key informants and stakeholders also reported that the WWNSF supported various native plant species such as *Podocarpus falcatus*, *Juniperus procera*, *Polyscias fulva*, *Olea capensis*, and *Rhus* species as well as wild animals such as Colobus, monkeys, and apes.

### 3.3. Estimated Provisioning FESs

The value of ESs comes from community-specific averages for the FES needed, combined with average market prices. This information is based on the consensus of the respondents. If there is no market price, participants must agree on a theoretical selling price for certain goods.  Table 4 shows the average and range of FESs values for provisioning goods, calculated using data from household respondents in the study area. Consequently, firewood had the highest price per household per year, followed by water supply for human and animal watering and timber production. On the other hand, the farming tool used for plowing (Eref) had the lowest value, with the horizontal beam (Mofer) and the yoke (Kenber) having slightly higher values, respectively. However, according to Krause et al. [33], freshwater and crops were the most important provisioning services that the local people obtained from the study area in the Oromia Region. This variation might be due to the fact that the estimation or valuation of the ESs conducted by Krause et al. [33] was derived from different land uses such as vegetative and cropland, which could encompass a very large area, where water and crop could be in abundance.

### 3.4. Total Estimated Provisioning FES

Each household sampled received an annual amount of provisioning FESs ranging from $206.74 to $1,957.67, with an average of $1,152.30. The forest-surrounding community also earned an average income of $1,152.30 per year, as the sample mean represents the population mean (Table 5). This implies that local communities get many additional incomes from the WWNSF, thus supporting their subsistence livelihoods, despite not yet understanding it well. Krause et al. [33] also confirmed that provisioning ESs is naturally and highly essential to people's livelihoods.

### 3.5. Monetary Estimates of WWNSF ESs

The FESs summarized in Table 6 are assigned a monetary value using the standard cost estimation method ($/ha) for tropical forest areas, based on the size of the WWNSF. Among the FESs, raw materials had the highest estimated value of $2,214,986, followed by erosion control at $1,722,767 and climate regulation at $1,568,069. Cultural services had the lowest estimated cost at $14,063.4, followed by genetic resources at $28,299.7. The total estimated cost of all FES amounted to be $14,112,622.00, which is high value generated from the WWNST. Similarly, according to Krause et al. [33], the highest TEV was obtained from forest ecosystems compared to each of the grassland and cropland ecosystems. If locals were enforced to pay this amount, it would be impossible or unaffordable. But, it is very essential to let them know the benefits of WWNST in terms of money, calculated here to make them realize and understand its uses, thus properly conserving and managing forests.

### 3.6. Regression Analysis

In Table 7, we encounter the R-value, a statistic similar to the correlation coefficient. This value stands as an indicator of the strength of the relationship between the two variables. Remarkably, the R-value in this study was 0.693, signifying a robust relationship and a well-performing predictive model. In addition, the R-squared value, represented as *R*^2^, explains 54% of the variation in the outcome variable (total FESs). Essentially, this means that 54% of the variance in the data can be attributed to the predictor variables. This highlights the importance of considering socioeconomic and demographic factors when designing forest management strategies and promoting sustainable forest use.

Table 8 explores the significance of the model, including independent variables, as a predictor of the outcome variable. This assessment was conducted through analysis of variance (ANOVA), resulting in a significance value of less than *p* ≤ 0.05 (specifically, *F* = 3.298, *p* ≤ 0.000). Consequently, we can assert with confidence that the regression model significantly predicts the overall FESs.

#### 3.6.1. Parameter Estimates

The findings regarding household characteristics offer valuable insights into forest dependency patterns (shown in Table 9). An interesting difference emerged when considering gender and forest dependency. Our study revealed that male-headed households were more involved in utilizing forest products (*β* = 24.10, *p* ≤ 0.019). In contrast, Garekae et al. [34] found women relying more heavily on forest resources due to traditional roles. This difference could be due to several factors specific to our study area, such as cultural norms or market access opportunities. Therefore, understanding how gender roles influence forest resource use is crucial to ensure equitable forest management practices [35].

Our findings revealed a positive and significant relationship between family size and the value of forest ecosystem services (FES) derived by the family (*β* = 362.94, *p* ≤ 0.000 and *β* = 207.56, *p* ≤ 0.013). This aligns with Project [36] and Tolera [37], who found that larger families collect more forest products, likely due to both increased labor availability and potentially higher consumption needs. This suggests the importance of considering household composition when evaluating forest resource-use patterns. Furthermore, other studies, such as Egoh et al. [38], suggest that strategies promoting sustainable forest use should consider the needs of larger families. There was an inverse correlation between the age of household heads and their dependence on forests. In other words, households led by younger individuals (aged 20–30 years) had a higher average dependence on forest resources compared to those led by older individuals (aged 46–60 years and over 60 years). This difference was statistically significant (*β* = −68.91, *p* ≤ 0.416). This finding aligns with research by Garekae et al. [34] and suggests a potential decline in resource collection efficiency with age. Older adults may be less able to efficiently collect, transport, and sell forest products compared to younger individuals. Furthermore, this study highlights the importance of considering factors such as household heads' health status and access to alternative income sources. These factors can also influence dependence on forest resources. Based on these findings, forestry programs could benefit from incorporating support mechanisms for older adults who rely on forest resources [39]. This could help ensure their well-being and promote sustainable forest management.

Our research found that education level did not necessarily decrease reliance on forest ecosystem services (FES). For example, households led by individuals with 5–8 years of education had an average of $227.38 higher dependence on FES compared to households with an illiterate head (*β* = 227.38, *p* ≤ 0.029). This aligns with the findings of Acharya et al. [40], who reported a positive association between education and willingness to pay for forest benefits. This could be due to limited alternative income opportunities in the study area, emphasizing the importance of considering local context when interpreting the impact of educational attainment on forest dependence [39].

The size of a household's landholding affects the annual value of the Forest Ecosystem Service (FES). Households with more farmland tend to have lower FES values. Specifically, households with larger landholdings exceeding 2 hectares have an FES value $28.55 lower than those with 0.5 hectares or less (*β* = −28.55, *p* ≤ 0.0065). This suggests that larger landholdings allow households to meet their needs through agriculture, thereby reducing their reliance on forest resources. This finding aligns with previous research by Sunderlin et al. [41].

While there was a negative correlation between off-farm activities and the annual value of forest resources collected (FES), this correlation was not statistically significant. In other words, households engaged in off-farm activities like trading, tree seed collection, security work, poultry farming, and beekeeping earned an average of $52.82 less per year from forest resources (*β* = −52.82, *p* ≤ 0.487) compared to those who did not participate in such activities. However, the specific type of off-farm activity might be important, with some activities potentially decreasing forest dependence more than others [42]. This aligns with research by Hong et al. [43] which suggests that diversifying income sources can lead to a reduced reliance on forest resources.

Multicollinearity tests were performed using the variance inflation coefficient (VIF) and error tolerance, as indicated in Table 9. If the VIF exceeds 10 or the tolerance falls below 0.1, it indicates the presence of severe multicollinearity, which requires correction. However, based on the current findings, these two values do not violate the assumption of multicollinearity (Table 9).

**Figure 2:**
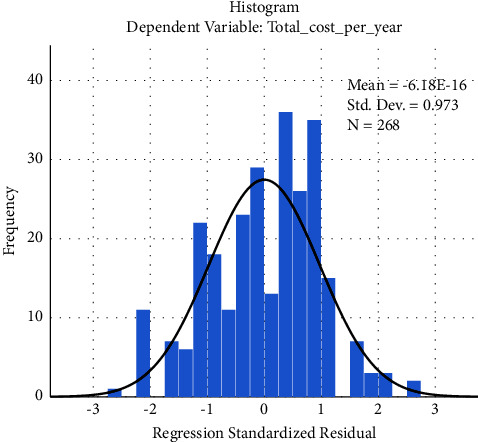
A histogram of the standardized residuals for the regression.

Test of the normality of residuals: The histogram indicates that the distribution did not exhibit longer tails compared to the normal distribution and did not violate the assumption of normality of residuals (Figure 2).

**Figure 3:**
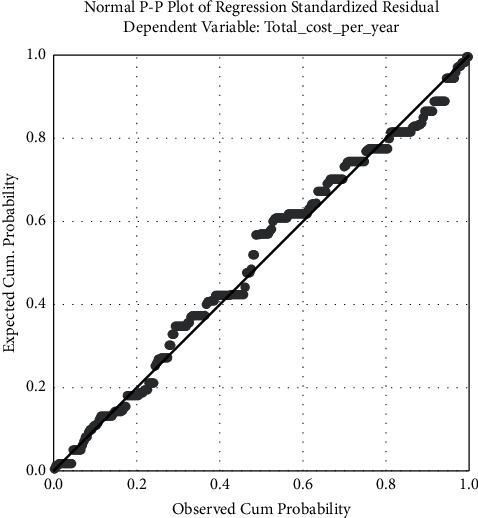
Normal probability plot (normal PP) of standardized residual.

Normal probability plot: The normality of the residuals can also be verified by examining the customary P-P plot of the normalized residuals of the regression. In Figure 3, it can be concluded that no point deviates significantly from the diagonal, indicating that the residuals conform to a normal distribution. Overall, the graph did not show any indications of anomalies, outliers, or unidentified variables (Figure 3).

**Figure 4:**
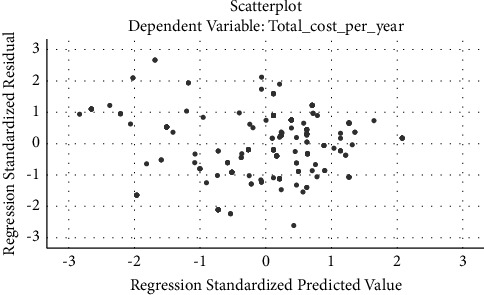
A plot of residual residuals versus predicted.

Residuals versus predicted (fits) plot: The plot of residuals versus predicted values shows a well-behaved plot. The points are randomly distributed around zero and do not show any systematic pattern. There are no clusters of points representing distinct groups in the data. In general, there are no discernible patterns in the residual plots, suggesting that a linear model is suitable for modeling these data (Figure 4).

### 3.7. Main Risk Factors for Forest Sustainability

**Figure 5:**
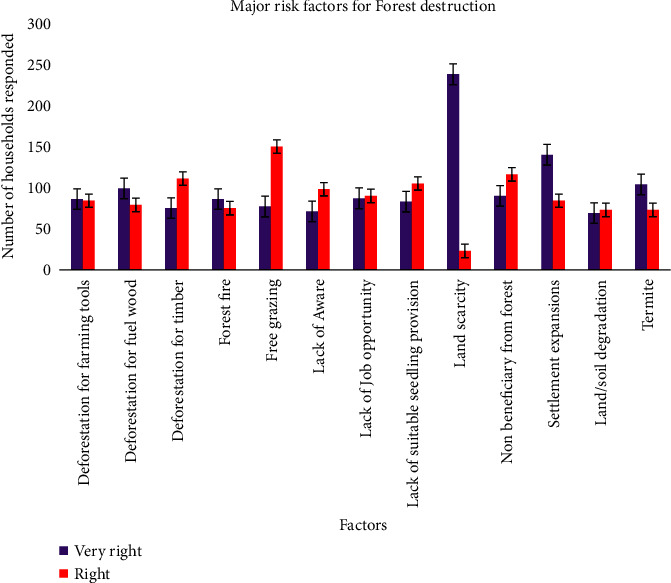
The main risk factors that influence the sustainability of the WWNSF.

Figure 5 presents the perceptions of the respondents about the main risk factors that affect forest sustainability, measured on a two-point scale ranging from very significant to significant. The results of the analysis indicate that the scarcity of farmland, the expansion of settlements, the logging of firewood and timber, and agricultural tools, as well as the lack of employment opportunities were the main drivers of forest degradation. Moreover, population growth, little or no alternative opportunities, and lack of sustainable land-use management are other challenges in Ethiopia [33] in general, and in the present study area, in particular. Arjjumend et al. [44] also reported similar results of the challenges encountered in Nechsar National Park of the country such as deforestation, lack of stakeholders' coordination, poverty, and lack of awareness about FESs derived in terms of monetary values.

### 3.8. Assessing Stakeholders and Their Roles

Based on the results of the WWNSF evaluation, the main stakeholders involved in direct and indirect conservation activities and beneficiaries were local communities, REDD+, Orthodox Development Organization, People for People organization, Environment and Wildlife Protection Office, Cooperative Societies at the Kebele level, Justice and Security organizations, and Culture and Tourism Office at the District and Zonal levels. Local communities were identified as the most important stakeholders, as they had a direct relationship with forest being beneficiaries and were also responsible for its protection and management. The Environmental and Wildlife Protection Offices and REDD+ of the Tarmaber and Ankober districts provided training, awareness, and employment opportunities to local communities to minimize the burden on WWNSF. These stakeholders also supported community livelihoods through initiatives such as providing livestock and assisting with seedling planting and re-afforestation programs. In addition, the Office of Justice and Security and Culture and Tourism played a role in resolving conflicts and promoting forest conservation. The Orthodox Development Organization provided basic consumable items to help farmers modernize agriculture. Similarly, the People for People Organization introduced modernizing agriculture and community life improvement packages, distributed fuel-efficient stoves, and more. Thus, the involvement and participation of the stakeholders are very vital for socioeconomic and ecological sustainability of the forest ecosystem [44].

### 3.9. Suggested Strategies

The stakeholders and informants expressed their concerns about the challenges of the forests and the endangered biodiversity. Therefore, they suggested strategies that can conserve forests and sustainably benefit the local community. Among the suggested strategies, raising awareness in the community, training and educating the stakeholders and selected individuals, developing guidelines and providing training, and protecting the forest from human and animal interference were forwarded. Other strategies included giving awards to those who are active in forest afforestation and protection, conducting research, preventing forest fires, and strengthening the governmental and nongovernmental institutions for generating their significant contributions, and finally to maintain the forest ecosystem health, thereby achieving the maximum FESs. Still, other strategies such as carbon trade or revenue and enrichment plantings of fast-growing endemic tree species to provide a sustainable fuel wood supply are crucial to increase benefits to the communities [45]. Ayenew and Tesfay [29] also recommended that awareness creation (to local communities) and capacity building in forest hydrology (to local experts), and understanding the relationship between water and forest ecosystem are very vital for a suitable use of forest resources. Moreover, it must be considered to improve the living conditions or provide alternatives to local rural communities that traditionally meet their needs for forest products as well as to develop trans-sectorial policies to harmonize Ethiopian land-use policies [33]. For enhancing the participation of the stakeholders in the sustainable management of the forest ecosystems, increasing public awareness and their participation through training and education, organizing annual events (such as Annual Tree Planting Day, Tree Day, World Environment day, Day for Biological Diversity, and the like), strengthening the roles of governmental and nongovernmental organizations, and enforcement of policy and legislation and regulation measures should be considered [44].

### 3.10. Strength and Limitations

This study combined valuation with socioeconomic survey to understand how the forest contributes to the community's well-being. It provided unique insights by linking the economic value of forest services to specific community characteristics, which is useful for sustainable management planning with community involvement. However, the study used cross-sectional study design that is unable to establish causality between socioeconomic factors and FES dependence. Moreover, the study relied on self-reported data for provisioning services, which might be biased, especially if respondents were hesitant due to law enforcement concerns. Still, our findings may not be directly generalizable to other forest ecosystems.

## 4. Conclusions

This study highlights the significant socioeconomic and ecological benefits provided by the WWNSF to surrounding communities. The forest offers a wide range of provisioning, regulating, and supporting services, generating substantial income for local households. The findings demonstrate a clear correlation between proximity to the forest and the level of resource extraction, with households closest to the forest and those facing limited agricultural opportunities relying more heavily on forest resources. However, the study also identifies potential threats to the forest's sustainability. Uncontrolled use and extraction of forest resources can lead to environmental degradation, including drought and soil erosion. To address these challenges, future forest management strategies should prioritize community involvement, explore alternative income sources (particularly for the younger generation), and promote stakeholders' collaboration. Furthermore, by valuing the full range of forest ecosystem services (FESs), decision-makers can make informed decisions regarding forest development and conservation. This study acknowledges limitations in capturing the complete value of FESs, particularly due to respondent hesitancy regarding certain forest products. Moreover, our findings may not be directly generalizable to other forest ecosystems. Therefore, future researches should focus on addressing these gaps and explore similar valuations in different regions [46, 47].

## Figures and Tables

**Table 1:** Number of respondents taken from the two districts with their respective kebeles with proportions of sex respondents.

District	Kebele	Household respondents				
		Male		Female		Total (%)
		Frequency	Percent	Frequency	Percent	
Tarmaber	Wof-Washa	85	89.5	10	10.5	95 (100)

Ankober	Laygorebela	45	93.8	3	6.3	48 (100)
	Mehal-Wonz	69	95.8	3	4.2	72 (100)
	Zego	47	88.7	6	11.3	53 (100)

Total		246	100	22	100	268 (100)

**Table 2:** A conceptual model of key components of the TEV concept [28].

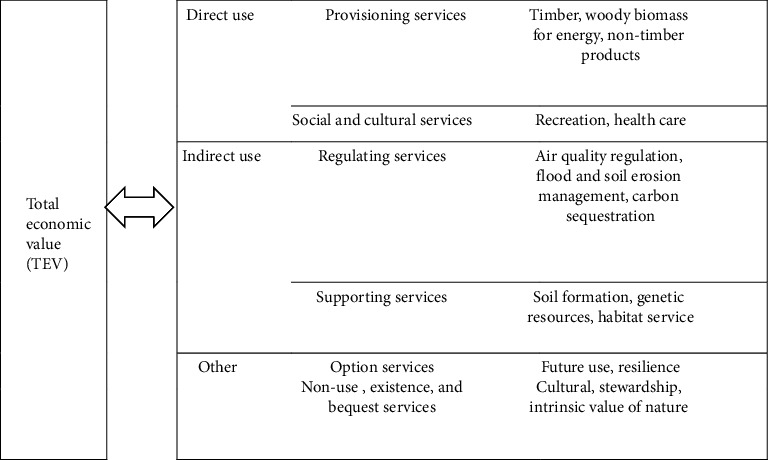

**Table 3:** Socioeconomic and demographic characteristics of the household and the head of the household.

Variables	Category	Frequency	Percent
Sex	Male	246	91.8
	Female	22	8.2

Age	20–30	65	24.3
	>30–45	69	25.7
	>45–60	113	42.2
	>60	21	7.8

Educational level	Illiterate	56	20.9
	Read and write	112	41.8
	1–4	26	9.7
	5–8	47	17.5
	9–12	27	10.1

Family size	1 to 2	32	11.9
	3 to 5	110	41.0
	6 and above	126	47.0

Land size	≤0.5 hectare	111	41.4
	0.51 to 1 hectare	119	44.4
	1.1 to 2 hectare	31	11.6
	>2 hectare	7	2.6

Off-farm activity	No	227	84.7
	Yes	41	15.3

Total		268	100.0

**Table 4:** Values of FES of WWNSF provisioning services per household per year.

Types of goods/services	ESs estimated cost ($/year)			
	Min.	Max.	Mean	Std. dev.
Water for human drinking	0.00	516.86	164.98	120.92
Water for animal drinking	0.00	861.44	322.72	201.04
Water for wash	0.00	56.60	22.73	12.32
Firewood	0.00	689.15	474.43	255.62
Timber	0.00	943.40	131.13	208.48
Farming tool for horizontal beam (Mofer)	0.00	16.51	5.11	3.68
Farming tool for yoke (Kenber)	0.00	18.40	6.91	5.00
Farming tool for handling (Eref)	0.00	5.19	0.65	1.15

**Table 5:** The annual total estimated value of the provisioning FESs per household.

	Min	Max	Mean	Std. dev.
Total cost per household	206.74	1,957.67	1,152.30	407.07

**Table 6:** Monetary estimated ecosystem values of WWNSF (tropical type) per year from the total area of the forest.

Ecosystem service	Market nature of the service	Global values by tropical forest type ($/ha)	Wof-Whasha forest ESs values ($/ha)
*Regulating services*			
Climate regulation	Nonmarket	223	1, 568, 069
Disturbance regulation	Nonmarket	5	35, 158.5
Water regulation	Nonmarket	6	42, 190.2
Water supply	Market, nonmarket	8	56, 253.6
Erosion control and sediment retention	Nonmarket	245	1, 722, 767

*Supporting services*			
Soil formation	Nonmarket	10	70, 317
Nutrient cycling	Nonmarket	922	6, 483, 227
Waste treatment	Nonmarket	87	611, 757.9

*Provisioning services*			
Food production	Market	32	225, 014.4
Raw materials	Market	315	2, 214, 986
Genetic resources	Market, nonmarket	41	288, 299.7

*Cultural services*			
Recreation	Market, nonmarket	112	787, 550.4
Cultural	Nonmarket	2	14, 063.4

Total		2,007	14, 112, 622

**Table 7:** The model summary to show model fitness and total variation explained in the model.

Model summary				
Model	*R*	*R* square	Adjusted *R* square	Std. error
1	0.693	0.54	0.108	384.56

**Table 8:** Analysis of variance to estimate testing the overall model.

Model	Sum of squares	Df	Mean square	*F*	Sig.
Regression	6,827,916.97	14	487,708.35	3.298	≤0.000
Residual	37,415,211.99	253	147,886.21		
Total	44,243,128.96	267			

**Table 9:** Households' socioeconomic and demographic factors influencing FESs of WWNSF.

Model	B	Std. error	T	Sig.	Tolerance	VIF
Constant	825.31	128.92	6.402	≤0.000		
Sex of HH^a^: female (ref^b^)						
Male	24.10	105.39	0.229	≤0.019	0.659	1.517
Family size: 1 to 2 (ref)						
3 to 5	362.94	91.10	3.985	≤0.000	0.275	3.638
6 and above	207.56	83.35	2.490	≤0.013	0.319	3.137
Age of HH: 20 to 30 (ref)						
31 to 45	65.61	96.11	0.683	0.495	0.245	4.082
46 to 60	−68.91	84.63	−0.814	0.416	0.403	2.481
>60	−46.12	130.78	−0.353	0.725	0.447	2.238
HH education level: illiterate (ref)						
Read and write	153.89	84.03	1.831	0.068	0.321	3.113
1 to 4	54.52	117.10	0.466	0.642	0.460	2.176
5 to 8	227.38	103.25	2.202	≤0.029	0.358	2.794
9 to 12	79.05	136.11	0.581	0.562	0.329	3.042
Land size: ≤0.5 hectare (ref)						
0.51 to 1 hectare	231.99	173.00	1.341	0.181	0.725	1.380
1.1 to 2 hectare	52.50	97.10	0.541	0.589	0.573	1.747
>2 hectare	−28.55	65.87	−0.433	≤0.006	0.515	1.941
Having off-farm: no (ref)						
Yes	−52.82	75.90	−0.696	0.487	0.739	1.353

^a^HH: household head; ^b^ref: reference.

## Data Availability

The data of this study are available in the manuscript and supplementary file.

## Supplementary Materials

Supplementary Materials Supplementary file: list of plant species identified from the five forest sites of North Shewa Zone, central Ethiopia (Sources: Simple survey of Wof Washa Forest, Teketay and Bekele [20]; Ayalew [22]).
