# Supplementary material for: Economic Valuation of Ecosystem Services and Its Association with Socioeconomic Factors in the Wof-Washa Natural State Forest, North Shewa Zone, Ethiopia
Source: Scientifica (Cairo). 2024 Jul 16;2024:6607551. doi: 10.1155/2024/6607551 (PMC11265944; doi:10.1155/2024/6607551)
Supplement: Supplementary Materials — Supplementary file: list of plant species identified from the five forest sites of North Shewa Zone, central Ethiopia (Sources: Simple survey of Wof Washa Forest, Teketay and Bekele [20]; Ayalew [22]). [file 6607551.f1.docx]

Supplementary file: List of plant species identified from the five forest sites of North Shewa Zone, central Ethiopia (Sources: Simple survey of Wof Washa Forest, Teketay and Bekele, 1995; Ayalew, 2018)

| no | Scientific name | Family | Local name (Amharic) | Habit |
| --- | --- | --- | --- | --- |
| 1 | *Acacia abyssinica* Hochst. ex Benth. | Fabaceae | Yabesha girar | T |
| 2 | *Acmella caulirhiza* Del. | Asteraceae | Yejoro guticha | H |
| 3 | *Allophylus abyssinicus* (Hochst.) Radlk. | Sapindaceae | Embus | T |
| 4 | *Aloe pulcherrima* | Aloaceae | Sete eret | S |
| 5 | *Aloe trichosantha* Berger | Aloaceae | Wende eret | S |
| 6 | *Andropogon abyssinicus* Fresen. | Poaceae | Gaya | T |
| 7 | *Asparagus africanus* Lam. | Asparagaceae | Serity | H |
| 8 | *Berberis holstii* Engl. | Berberidaceae | Zyrintila/zinkila | S |
| 9 | *Bersama abyssinica* Fresen. | Melianthaceae | Azamir | T |
| 10 | *Bidens pilosa* L. | Asteracea | Chogogit | H |
| 11 | *Buddleja polystachya* Fresen. | Loganiaceae | Anfar | T |
| 12 | *Calpurnia aurea* (Ait.) Benth. | Fabaceae | Digita | S |
| 13 | *Carissa spinarum* L. | Apocynaceae | Agam | S |
| 14 | *Cassipourea malosana* (Baker) Alston | Rhizophoraceae | Werer | T |
| 15 | *Caucanthus auriculatus* Radlk.) Nie Denzu | Malpighaceae | Tota kula | L |
| 16 | *Celtis africana* Burm.f. | Ulmaceae | Qewt | T |
| 17 | *Clematis hirsuta* Perr. and Guill. | Ranunculaceae | Azo hareg | L |
| 18 | *Clerodendrum myricoides* (Hochst.) Vatke | Lamiaceae | Misirich | S |
| 19 | *Clutia abyssinica* Jaub.and Spach. | Euphorbiaceae | Fiyele fej | S |
| 20 | *Colutea abyssinica* Kunth and Bouche | Fabaceae | Duaduatie | S |
| 21 | *Crinum abyssinicum* Hochst. ex A.Rich. | Amaryllidaceae | Yejib shinkurt | H |
| 22 | *Croton macrostachyus* Del. | Euphorbiaceae | Bisana | T |
| 23 | Cyphostemma cyphopetalum | Vitaceae | Gindosh | H |
| 24 | *Debregeasia saeneb* (Forssk.) Hepper & Wood | Urticaceae | Wenz Admik | S |
| 25 | *Discopodium penninervium* Hochst. | Solanaceae | Ameraro | S |
| 26 | *Dodonea angustifolia* L.F. | Sapindaceae | Kitkita | S |
| 27 | *Dombeya torrida* (J.F. Gmel.) P. Bamps | Sterculiaceae | Wulkifa | S |
| 28 | *Dracaena fragrans* (L.) Ker Gawl. | Dracenaceae | Merqo |  |
| 29 | *Dregea rubicunda* K. Schum. | Asclepiadaceae | Qwando hareg | L |
| 30 | *Echinops longisetus* A. Rich | Asteraceae | Koshele tiliqu | S |
| 31 | *Embelia schimperi* Vatke | Myricaceae | Enqoqo | L |
| 32 | *Erica arborea* L. | Ericaceae | Asta | S |
| 33 | *Erythrococca trichogyne* (Muell. Arg.) Prain | Euphorbiaceae | Shiro |  |
| 34 | *Euclea racemosa* Murr. |  | Dedeho | S |
| 35 | *Ferula communis* L. | Apiaceae | Qereshashmbo | H |
| 36 | *Galiniera saxifraga* (Hochst.) Bridson | Ruiaceae | Tota Qula | S |
| 37 | *Galinsoga parviflora* Cav. | Asteraceae | Deha neqel | H |

Supplementary file: Continued……

| no | Scientific name | Family | Local name (Amharic) | Habit |
| --- | --- | --- | --- | --- |
| 38 | Gladiolus abyssinicus (Brongn. Ex Lemaire)Goldblattandde Vos | Iridaceae | Yejib shinkurt tinshu | H |
| 39 | *Glycine wightii* (wight and Arn.)Verdc. | Fabaceae | Yayit hareg | L |
| 40 | *Halleria lucida* L. | Scrophulariaceae | Mesenqero | T |
| 41 | *Hyparrhenia hirta* (L.) Stapf | Poaceae | Senbelet | H |
| 42 | *Hyparrhenia nyassae* (Rendle) Stapf | Poaceae | Senbelet qechinu | H |
| 43 | *Hypericum revolutum* Vahl | Hypericaceae | Amja | T |
| 44 | *Indigofera spicata Forssk.* | Fabaceae | Gerengere |  |
| 45 | *Inula confertiflora* A. Rich.** | Asteracea | Weynagift |  |
| 46 | *Ipomoea tenuirostris* Choisy subsp.tenuirostris Choisy | Convolvulaceae | Yayit hareg | H |
| 47 | *Jasminum abyssinicum* Hochst.ex DC. | Oleaceae | Abita | L |
| 48 | *Jasminum grandiflorum* L. subsp. *Floribundum* (R. Br. Ex Fresen.) | Oleaceae | Tenbelel | L |
| 49 | *Juniperus procera* Hochst. ex Endl. | Cupressaceae | Yabesha tid | T |
| 50 | *Kalanchoe petitiana* A. Rich | Crassulaceae | Endehuahula | H |
| 51 | *Laggera tomentosa* (Sch.Bip. ex A. Rich.)Oliv.andHiern | Asteraceae | Keskeso | S |
| 52 | *Lippia adoensis* Hochst.ex Walp. | Verbenaceae | Koseret/Kessie | S |
| 53 | *Lobelia gibberroa* Hemsl. | Lobeliaceae | Jibira | H |
| 54 | *Maesa lanceolata* Forssk. | Myrsinaceae | Kelewa | T |
| 55 | *Maytenus arbutifolia* (A. Rich.) Wilczek | Celastraceae | Atat | T |
| 56 | *Maytenus undata* (Thunb.) Blakelock | Celastraceae | Damot Weyra | T |
| 57 | *Momordica foetida* Schumach. | Cucurbitaceae | *Yamora misa* | S |
| 58 | *Myrica salicifolia* A.Rich. | Myricaceae | Shinet | T |
| 59 | *Myrsine africana* L. | Myrsinaceae | Qechemo | S |
| 60 | *Nuxia congesta* R. Br. ex Fresen. | Loganiaceae | Askuwar | T |
| 61 | *Olea capensis* L. subsp. *macrocarpa* (C.H. Wright) Verdc. | Oleaceae | Wegeda | T |
| 62 | *Olea europaea* L.subsp. cuspidata (Wall.ex G. Don.) Cif. | Oleaceae | Weyra | T |
| 63 | *Olinia rochetiana* A. Juss. | Oliniaceae | Tife | T |
| 64 | *Osyris quadripartita* Decn. | Santalaceae | Keret | S |
| 65 | *Otostegia fruticosa* (Forssk.) Schweinf. ex Penzig subsp. Fruticosa | Lamiaceae | Geram tunjit | S |
| 66 | *Otostegia tomentosa* A. Rich. | Lamiaceae | Kese-sendel | S |
| 67 | *Phytolacca dodecandra* L 'Herit. | Phytolaccaceae | Endod | L |
| 68 | *Pittosporum viridiflorum* Sims | Pittosporaceae | Weyil | T |
| 69 | *Plectocephalus varians* *(A. Rich.)* C. *JefJreyex Cufod.* | Asteraceae | Koshele tinshu | H |

Supplementary file: Continued……

| 70 | Podocarpus falcatus (Thunb.) R.B ex Mirb. | Podocarpaceae | Zigba | T |
| --- | --- | --- | --- | --- |
| 71 | *Polygala steudneri* Chod. | Polygalaceae | Etse libona | H |
| 72 | *Polyscias fulva* (Hiern) Harms | Araliaceae | Yeznjero wenber | T |
| 73 | *Premna schimperii* Engl. | Lamiaceae | Chocho | S |
| 74 | *Prunus africana* (Hook.f.) Kalkm. | Rosaceae | Tikur enchet | T |
| 75 | *Psydrax schimperiana* (A. Rich.) Bridson | Rubiaceae | Seged | T |
| 76 | *Pterolobium stellatum* (Forssk.) Brenan | Fabaceae | Kontir | L |
| 77 | *Rhoicissus tridentata* (L.f.) Wild & Drumm. | Vitaceae | Jenfoq | L |
| 78 | *Rhus natalensis* Krauss | Anacardiaceae | Busi | T |
| 79 | *Rhus retinorrhoea* Oliv. | Anacardiaceae | Tilem | T |
| 80 | *Rhus vulgaris* Meikle | Anacardiaceae | Qimo | T |
| 81 | *Rosa abyssinica* Lindley | Rosaceae | Kega | S |
| 82 | *Rumex abyssinicus* Jacq. | Polygonaceae | Meqmeqo | H |
| 83 | *Rumex nepalensis* Spreng. | Polygonaceae | Tult | H |
| 84 | *Rumex nervosus* Vahl | Polygonaceae | Embuacho | S |
| 85 | *Salvia nilotica* Jacq. | Lamiaceae | Hulegeb | H |
| 86 | *Schefflera abyssinica* (Hochst. Ex A. Rich.) Harms | Araliaceae | Gitem |  |
| 87 | *Sida schimperiana* Hochst. Ex A. Rich. | Malvaceae | Chifirig | S |
| 88 | *Snowdenia petitiana* (A. Rich.) CE Hubbard | Poaceae | Qua senbelet | H |
| 89 | *Solanecio gigas* (Vatke) C. Jeffery | Asteraceae | Shikoko gomen | S |
| 90 | *Solanum incanum* L | Solanaceae | Yemidr Enbuay | S |
| 91 | *Solanum marginatum* L.f. | Solanaceae | Embuay | S |
| 92 | *Stephania abyssinica* (Dillon and A. Rich.) Walp. | Menispermaceae | Kelala/Engochit hareg | H |
| 93 | *Thymus schimperi* Ronnigersubsp*.schimperi* | Lamiaceae | Tosign | H |
| 94 | *Trichocladus ellipticus* Eckl. & Zeyh. | Hamamelidaceae | Abilwuha | T |
| 95 | *Trifolium calocephalum* Fresen. | Fabaceae | Maget | H |
| 96 | *Turraea holstii* Gurke | Meliaceae | Atesa | S |
| 97 | *Vernonia auriculifera* Hiern | Asteracea | Gujjo | S |
| 98 | *Zehneria scabra* (Linn.f.) Sond. | Cucurbitaceae | Yayithareg | H |
